# Using nanopore sequencing to identify bacterial infection in joint replacements: a preliminary study

**DOI:** 10.1093/bfgp/elae008

**Published:** 2024-03-30

**Authors:** Hollie Wilkinson, Jamie McDonald, Helen S McCarthy, Jade Perry, Karina Wright, Charlotte Hulme, Paul Cool

**Affiliations:** Centre for Regenerative Medicine, School of Pharmacy and Bioengineering, Keele University, Keele, UK; Oswestry Keele Orthopaedic Research Group (OsKOR), The Robert Jones and Agnes Hunt Orthopaedic Hospital Foundation Trust, Oswestry, UK; School of Biosciences, Cardiff University, Cardiff, UK; Centre for Regenerative Medicine, School of Pharmacy and Bioengineering, Keele University, Keele, UK; Oswestry Keele Orthopaedic Research Group (OsKOR), The Robert Jones and Agnes Hunt Orthopaedic Hospital Foundation Trust, Oswestry, UK; Centre for Regenerative Medicine, School of Pharmacy and Bioengineering, Keele University, Keele, UK; Oswestry Keele Orthopaedic Research Group (OsKOR), The Robert Jones and Agnes Hunt Orthopaedic Hospital Foundation Trust, Oswestry, UK; Centre for Regenerative Medicine, School of Pharmacy and Bioengineering, Keele University, Keele, UK; Oswestry Keele Orthopaedic Research Group (OsKOR), The Robert Jones and Agnes Hunt Orthopaedic Hospital Foundation Trust, Oswestry, UK; Centre for Regenerative Medicine, School of Pharmacy and Bioengineering, Keele University, Keele, UK; Oswestry Keele Orthopaedic Research Group (OsKOR), The Robert Jones and Agnes Hunt Orthopaedic Hospital Foundation Trust, Oswestry, UK; School of Medicine, Keele University, Keele, UK; Oswestry Keele Orthopaedic Research Group (OsKOR), The Robert Jones and Agnes Hunt Orthopaedic Hospital Foundation Trust, Oswestry, UK; School of Medicine, Keele University, Keele, UK

**Keywords:** genomic sequencing, bacteria, prosthetic joint infections, surgical infections, antibiotic resistance, nanopore

## Abstract

This project investigates if third-generation genomic sequencing can be used to identify the species of bacteria causing prosthetic joint infections (PJIs) at the time of revision surgery. Samples of prosthetic fluid were taken during revision surgery from patients with known PJIs. Samples from revision surgeries from non-infected patients acted as negative controls. Genomic sequencing was performed using the MinION device and the rapid sequencing kit from Oxford Nanopore Technologies. Bioinformatic analysis pipelines to identify bacteria included Basic Local Alignment Search Tool, Kraken2 and MinION Detection Software, and the results were compared with standard of care microbiological cultures. Furthermore, there was an attempt to predict antibiotic resistance using computational tools including ResFinder, AMRFinderPlus and Comprehensive Antibiotic Resistance Database. Bacteria identified using microbiological cultures were successfully identified using bioinformatic analysis pipelines. Nanopore sequencing and genomic classification could be completed in the time it takes to perform joint revision surgery (2–3 h). Genomic sequencing in this study was not able to predict antibiotic resistance in this time frame, this is thought to be due to a short-read length and low read depth. It can be concluded that genomic sequencing can be useful to identify bacterial species in infected joint replacements. However, further work is required to investigate if it can be used to predict antibiotic resistance within clinically relevant timeframes.

## BACKGROUND AND INTRODUCTION

### Introduction

This study aims to determine if nanopore sequencing can correctly identify the species of bacteria causing prosthetic joint infections (PJIs) from prosthetic fluid samples collected at the time of revision surgery. In addition, it is investigated if the speed of identification can be improved using this technique. The nanopore sequencing in this study uses the MinION device from Oxford Nanopore Technologies (ONTs), which is a cost-effective method for genomic sequencing that can be used ‘in the field’ compared to other nanopore devices available such as PromethION [1]. Microbiological cultures are the gold standard for diagnosing PJI. This investigation compares the results from nanopore sequencing to microbiological cultures. The samples utilized in this study were collected and processed in the hospital where patients are being treated for PJI. MinION nanopore sequencing is suitable to be used in the hospital environment. Other sequencing techniques, such as Illumina sequencing, would be more difficult to perform in the hospital environment and take longer to produce results. This paper addresses the current challenges of diagnosing PJI including false-negative results, delays from sample collection and how nanopore sequencing may offer an opportunity to overcome these challenges [2].

### Clinical indications of PJI

Often the first indication of PJI is clinical presentation, including the joint being painful, inflamed, swollen, red and discharge from the wound. Clinical signs are usually followed up by further investigations including blood biochemistry, microbiological cultures and histological review [2].

### Haematology and biochemistry

Biochemical markers are cheap to obtain and can support the suspicion of infection [3]. Inflammatory markers including C-reactive protein (normal value <5 mg/L) and erythrocyte sedimentation rate (ESR, normal value <10 mm/h) are often raised in response to an infection [2]. ESR can often be raised due to inflammatory conditions, including inflammatory arthropathy or rheumatoid arthritis. This needs to be taken into consideration when interpreting biochemical results with regards to infection. Furthermore, these biochemical markers can also be raised due to the normal response to surgery, making interpretation with regards to infection difficult. Sequential tests can help, with a downward trend following surgery being reassuring that there is no ongoing infection. An increased white cell count in the blood or synovial fluid from a prosthetic joint can also support a diagnosis of PJI [4].

### Other tests

Synovasure is a point of care test used to rule out PJI during revision surgery. Synovasure is a lateral flow type test to detect the presence of alpha-defensin in a sample of prosthetic fluid. Alpha-defensin is an antimicrobial peptide that is released in response to invading pathogens. This test has been reported to be useful for ruling out PJI when negative [5].

### Microbiological cultures

Bacterial identification is required to guide antibiotic strategy. Currently, microbiological cultures are used to identify the species of bacteria causing PJI. Samples of prosthetic fluid and/or tissue are obtained during revision surgery and cultured in the laboratory. This process takes at least 48 h and often longer. Bacterial identification is required to guide an antibiotic strategy. Microbiological cultures have many limitations and can produce false-negative results (no growth), particularly if antibiotics were administered before samples were taken [6]. Increasing culture incubation time can reduce the number of false-negative results; however, this prolongs the time from surgery to treatment with appropriate antibiotics. Furthermore, it increases the risk of false-positive results (i.e. contamination). Contamination can occur when the sample is collected during surgery or during subsequent handling in the laboratory. Contaminant bacteria are often skin flora or from the environment where the samples were processed. Collecting multiple samples makes false-positive results, due to contamination, easier to identify. It is recommended to take at least five samples for microbiological cultures [7]. Microbiological cultures are also used to identify antibiotic resistance in bacteria causing PJI. However, investigating antibiotic resistance in this way takes a considerable amount of time and relies on the initial culture identifying the bacteria species causative of the infection.

### Nanopore sequencing with the MinION

ONTs have a range of library preparation kits available for genomic sequencing depending on the users’ intentions. For this study, the ‘Rapid Sequencing’ kit (SQK-RAD004) was selected as it is designed for fast library preparation and producing long-reads. The protocol provided by ONT for preparing the DNA library and genomic sequencing was used in this study [1]. The MinKNOW software from ONT generates fast5 files that are translated to fastq files using a given basecaller. The ONT software suite includes a basecaller called guppy. Fastq files contain the nucleotide sequence as well as a quality parameter for each base that has been called. The quality score for each base in the fastq file is indicated by an associated phred score (logarithmically related to the probability of error), which is represented by an American Standard Code for Information Interchange character [8]. This score is useful to filter genomic data, so that only high-quality data are included in subsequent analysis.

Some genomic classification tools, including Basic Local Alignment Search Tool for Nucleotides (BLASTN), require sequencing data in fasta format. Fasta files are similar to fastq files, but without the quality scores [9]. Fastq files can be converted to fasta files by removing the quality scores.

### Genomic sequencing to identify bacteria

There are several examples in the literature demonstrating the use of nanopore sequencing to identify the species of bacteria causing infections. Examples include respiratory infections [10], blood stream infections [11] and orthopaedic infections [12]. Current work demonstrates that investigations with nanopore sequencing can generate results in real-time, faster than other genomic sequencing techniques such as Illumina sequencing [12]. Genomic sequencing has been demonstrated to be useful in identifying bacteria in cases of PJI when culture results are negative, this may be due to the species of bacteria being difficult to grow in microbiological cultures [13]. Concerns for the use of genomic sequencing to identify infection causing bacteria include false positives due to sample contamination and the identification of bacteria that may be part of the normal flora [13]. Nanopore sequencing is reported to have lower accuracy in comparison to other genomic sequencing techniques but has reduced costs and a quicker turn-around time to results [13]. Genomic sequencing of the 16S rDNA region of bacterial genomes has also been identified as a method to identify bacterial species. The 16S region is a common region of all bacterium genomes but differs enough between species to differentiate between them. This method, whilst useful for species identification, does not provide information about other genes such as antibiotic resistance genes [14].

### Antibiotic resistance

Antimicrobial resistance (AMR) genes impart microbes with antibiotic resistance mechanisms, causing increased risk to the patient. Furthermore, there is risk of the spread of AMR genes between microbes in a healthcare environment, as there are opportunities for DNA exchange to occur between bacteria [15]. This is due to bacterium’s ability to transfer genes from one bacterium to another via several processes such as conjugation, transduction and transformation [16].

### Antibiotics resistance gene databases

There are a range of open access genomic databases available with a collection of identified resistance genes. The three largest AMR gene databases are: NCBI Pathogen Detection Reference Gene Catalogue (which uses AMRFinderPlus as a tool to search the database), ResFinder [17] and Comprehensive Antibiotic Resistance Database (CARD), which uses the Resistance Gene Identifier (RGI) programme [18].

CARD is a database of AMR genes, associated proteins and resistance phenotypes. To run genomic sequence classification using this database, the genomic sequencing data must be in fasta format. The software is used to compare genomic sequencing data to a database of AMR genes using the RGI. RGI filters reads by removing short sequences and predicts associated proteins with a specified confidence level [16]. This database may not be appropriate to use clinically as only described mutations associated with AMR can be identified [19].

ResFinder is a classification programme that uses partial and complete genomes to identify the presence of antimicrobial-resistant genes and accepts either fastq or fasta files. Classification uses the reference sequences present in the ResFinder database. Matches between the query sequences and reference database are identified with a default threshold of a 100% identity and therefore are reliable for the identification of AMR genes [19].

## METHODS AND RESULTS

Samples of prosthetic fluid were taken during revision surgery or aspiration from patients with known PJI. A group of patients identified during the study period with normal blood test results and no clinical indication of infection acted as negative controls. Informed consent was obtained from every patient before samples were collected.

### Data collection

All samples were recorded in a database and pseudo-anonymized with a study number. The database is housed on a backed-up hospital server and contains the only key to patient identification. Sample type and location were also recorded on the database according to Human Tissue Authority regulations. The study has been approved by the Health Research Authority (20/HRA/4857). Blood test, microbiological and histopathological results were also recorded and made available to the researchers.

### Sample collection and DNA extraction

Samples of prosthetic fluid were taken from patients with PJI during revision surgery or aspiration (*n* = 10). A group of patients identified during the study period with normal blood test results and no clinical indication of infection acted as negative controls. To investigate if it was possible to identify AMR genes using nanopore sequencing, one patient with a confirmed *Staphylococcus aureus* infection resistant to erythromycin was identified. Prosthetic fluid samples were collected in the ultra-clean air operating theatre in sterile pots.

Samples were immediately taken to the laboratory and the DNA was extracted with the MagAttract kit from Qiagen, as recommended by ONT [20, 21]. The Qiagen MagAttract DNA blood kit uses a magnetic base and beads for the extraction of DNA from samples. The extracted DNA can be used immediately for most methods of genomic sequencing [22].

### DNA quantification

The DNA was quantified using an LVis microplate, read on a microplate reader (FluoStar Omega, BMG Labtech). The LVis microplate measures the amount of light passing through the sample against a reference value [23]. Subsequently, the total quantity of DNA in the sample is calculated. The ratio of the optical sample density at 260 nm to that at 230 nm is also measured. This value (A260/A230) should be approximately 1.8 for DNA samples. High variation from this value suggests that the sample may be contaminated and is not suitable for genomic sequencing [22].

### Nanopore sequencing

Once the DNA extraction was complete, the samples were sequenced using nanopore sequencing. For successful nanopore sequencing, a minimum of 400 ng of DNA is required [6]. The DNA library was prepared using the Rapid Sequencing Kit, following the protocol from ONT [1]. The quantification of DNA obtained from LVis microplate analysis was used to calculate the volume of the DNA library used in the protocol [23]. Sequencing was then performed with a standard flow cell for 120 min to produce an abundance of data. Basecalling was performed using the guppy basecaller (ONT) to produce fastq files, which were converted to fasta files with biopython’s SeqIO function [24].

### Determination of optimum genomic sequencing time

Preliminary investigations were performed to determine the minimum sequencing time needed to detect the species of bacteria causing the infection. DNA was extracted from the prosthetic fluid of a patient with known *S. aureus* infection following a total knee replacement. The DNA was then sequenced using the MinION for 2 h, and basecalling was performed using guppy basecaller (ONT). This produced 53 fastq files of a total of 533 MB of data. Table 1 shows the quantity of sequencing data produced using nanopore sequencing from a patient. The resultant fastq files were concentrated to represent different sequencing times. For example, the first seven fastq files were combined to represent 15-min sequencing time with a confirmed *S. aureus* infection for different theoretical sequencing times.

**Table 1:** The quantity of data and the theoretical sequencing time from nanopore sequencing and indication if this quantity of data was able to detect the Staphylococcus aureus genome in a patient with a confirmed Staphylococcus aureus infection

Data quantity (MB)	Sequencing time represented (min)	*Staphylococcus aureus* detected? (Y/N)
533	120	Y
267	60	Y
133	30	Y
67	15	Y

The combined fastq files were converted to fasta format, and classification was done using BLASTN*.*

Based on these results, it can be concluded that 15-min sequencing time seems sufficient for identification of bacterial genomes for the purposes of this study.

### Quality control

**Figure 1:**
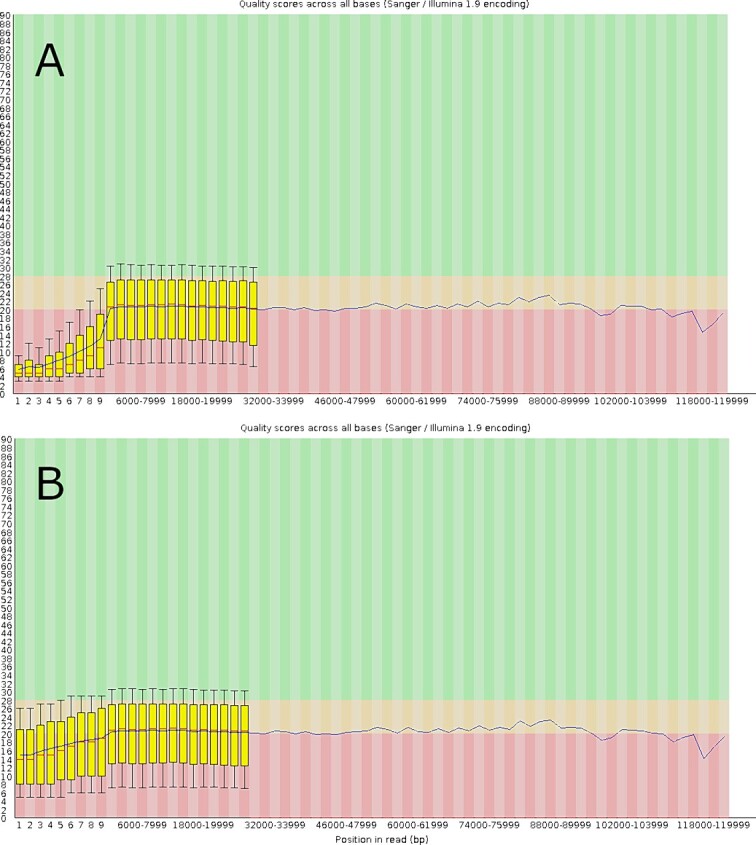
Phred quality scores of nanopore sequencing reads before (**A**) and after (**B**) trimming with Porechop and Nanofilt.

Initial quality assessment of the sequencing data was performed using the summary statistics produced during sequencing (example in Appendix 1). The adaptor sequences added during the preparation for nanopore sequencing were identified and removed with the Porechop application which is specifically designed to remove adapters added during nanopore sequencing [25]. Porechop’s default settings were used and set to remove adapter sequences with identity score of at least 90%. Then, poor-quality reads were trimmed with Nanofilt [26]. Nanofilt was used to remove the first 10 and last 10 bases from each sequence as these are usually poor quality. Reads with a quality score less than 85 and length of less than 100 were removed. Figure 1 shows the improvement in quality following quality control with Porechop and Nanofilt.

### Development of pipelines for genomic sequence classification

Once the genomic sequencing data were produced, the sequences were classified using a genomic classification programme to identify what species genomes are present. There are several genomic classification programmes available, and different programmes were investigated.

BLASTN is the current ‘gold standard’ for genomic sequence classification but is slower than other classification programmes that are available such as Kraken2 and MINDS (which uses the centrifuge algorithm) [22]. Sequencing data from the patient with a confirmed *S. aureus* infection were classified using BLASTN, Kraken2 and MINDS. The results were compared to determine if *S. aureus* could be detected and how long the classification process took. The combined file representing 15 min of sequencing time was chosen for this investigation, and the results are presented in Table 2. Only BLASTN and Kraken2 identified *S. aureus*. Kraken2 detected a greater number of species than BLASTN but the speed of both classification tools was similar and sufficient for this study.

**Table 2:** Results from the preliminary investigations of which genomic classification tool would be most suitable for use in this experiment

Classification tool	Detection of *Staphylococcus aureus*?	Time to classify (seconds)	Number of species detected
BLASTN	Yes	213	205
Kraken2	Yes	109	840
MINDS	No	23	165

When classifying the genomic sequences using BLASTN, filters were applied to only include sequences with an identity score of 90 or more. Sequence classification with BLASTN only used the bacterial genome database (taxid = 2) [22] as this makes classification faster than using the entire database for all organisms and excludes any results that are not bacteria. The BLASTN output was set to include the mean length, mismatch, identity score, gap score and e-value for each hit in the results file in comma-separated values (csv) format.

Based on the results from this preliminary analysis, BLASTN or Kraken2 are both suitable for identification of bacteria in PJI. Although Kraken2 is faster, this speed difference has no consequence for the purpose of this study. BLASTN is the accepted gold standard for genomic sequence classification as it uses a genome-wide approach to classification, whereas Kraken2 uses a k-mer-based approach. Furthermore, several studies have reported higher accuracy in genomic sequence classification using BLAST over Kraken2 and was taken forward as the preferred approach in this study.

Once classification was complete, the output was further analysed in R (version 4.3.1). The results from classification using BLASTN were filtered using a predetermined list of species associated with PJI (Appendix 2). This aims to exclude any species identified which are not pathogenic and would be associated with the healthy flora or may have contaminated the sample during collection or preparation. The remaining hits were then sorted by ascending e-value. The e-value indicates the probability that the nucleotide sequence was assigned to that organism by chance, with a lower e-value indicating a higher quality result [27]. The workflow from sample collection to results is given in Appendix 3 and versions of used software in Appendix 4.

The programmes CARD, AMRfinder and ResFinder were not able to identify any AMR genes from the genomic sequencing data produced in this study. Other studies have been able to detect antibiotic resistance genes using AMRFinderPlus and the rapid sequencing kit (SQK-RAD004) but after sequencing for 48 h [28]. This suggests that the required read length and depth were not reached by a 15-minu sequencing run. In future, AMR gene identification will be re-attempted using an increased run time or alternative library preparation kit.

### Genomic sequencing to confirm infections identified using microbiology

To date, 52 samples have been collected for this study with 33 confirmed or strongly suspected to have PJI and 23 with a positive microbiology result. Ten samples from patients with confirmed PJI have been sequenced using nanopore sequencing (15-min sequencing time), and the results are summarized in Table 3. Nanopore sequencing detected the species identified by microbiological culture in all but one of the samples analysed, although it did produce several potential false-positive results for each sample (where species have not been detected using standard microbiology). Further work to improve the bioinformatic techniques to reduce the rate of false-positive hits will be necessary.

**Table 3:** A summary of the samples that have been sequenced using nanopore sequencing following a positive microbiology result

Sample ID	Microbiology results	Detected using genomics? (Y/N)	Median e-value
4	*Staphylococcus aureus*	Y	1.4e^−68^
16	*Pseudomonas* species	Y	1.06e^−57^
18	*Escherichia coli*	Y	1.3e^−50^
26	*Enterococcus faecalis*	Y	1.24e^−26^
28	*Staphylococcus aureus*	Y	4.8e^−50^
29	*Staphylococcus aureus*	Y	5.9e^−73^
31	*Staphylococcus capitis*	Y	1.6e^−15^
31	*Staphylococcus epidermis*	Y	7.4e^−51^
37	Coagulase negative *Staphylococcus*	Y	1.9e^−53^
40	*Staphylococcus aureus*	Y	6.8e^−56^
40	Diptheroids	N	n/a
46	*Staphylococcus aureus*	Y	4.7e^−64^

**Figure 2:**
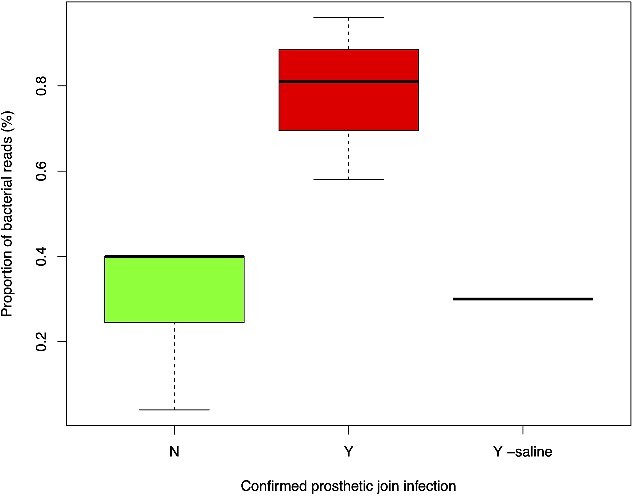
Boxplot showing the difference in the proportion of nanopore sequencing reads assigned to bacteria from patients with PJI (Y) and patients without PJI (N). One patient with confirmed PJI had saline injected into the joint before to aspiration (Y-saline).

Following classification, the proportion of total reads assigned to bacteria for each sample was calculated and expressed as a percentage. The proportion of reads assigned to bacteria for patients with confirmed PJI and without PJI are shown in Figure 2. One patient who did not have free fluid around the prosthesis had Normal Saline injected into the joint before aspiration. This technique is known to cause false-negative results for microbiological cultures due to a low concentration of bacteria in the sample. Consequently, this patient was analysed separately.

Figure 2 indicates that a higher quantity of reads was assigned to bacteria in samples from patients with confirmed PJI than from those without PJI. A Wilcox rank sum test confirms a statistically significant difference (*P* = 0.025).

## DISCUSSION

The results from this study suggest that the identification of the species of bacteria causing PJI is possible using nanopore sequencing. The identification in a short sequencing time suggests that nanopore sequencing could be useful in improving the current speed of diagnosis of PJI. There are several advantages of using this technique over microbiological cultures, including the identification of difficult to culture bacteria. Also, in cases of polymicrobial infections where current culture techniques often fail to identify multiple species due to competition between bacteria in culture or different growth requirements, nanopore sequencing has the potential to overcome these challenges [7].

Although identification of bacteria seems possible using the methods described, the identification of antibiotic resistance genes may need an extended sequencing time to produce more or longer reads. The bioinformatic programmes used to identify antibiotic resistance genes in genomic sequencing data often have a minimum read length and read depth for successful identification. It may be worth considering the use of other library preparation kits from ONT such as the ‘ligation sequencing kit’ as this kit is designed to produce longer reads. However, this kit requires a longer sample preparation time compared to the ‘rapid sequencing kit’ [29]. Even with longer sample preparation times, this method would still be considerably quicker than the extended microbiological cultures used to detect antibiotic resistance.

This investigation was performed on a limited number of patients and failed to identify the species of bacteria identified by microbiological cultures in one patient. Nanopore sequencing also identified several other species in each sample but further work into identifying contaminant species and minimum thresholds to identify a species as causative of PJI should overcome this challenge. Misclassification will always be a challenge with genomic sequencing data due to the high level of similarity between bacterial genomes. Genomic classification programmes (BLAST) often assign multiple species to a single genomic sequence [30]. Many species share large proportions of their genome, and individuals within a population vary from each other and the species reference genome due to natural variation caused by mutational events. This means there will never be a perfect match between all genomic sequencing data and a reference genome used for sequence classification purposes. As nanopore sequencing has a higher error rate than other genomic sequencing techniques, this may increase the risk of multiple species being assigned to a single read and incorrect classification. However, the huge benefits of nanopore sequencing with regards to cost, time and ease of use make it a valuable method to be considered for clinical applications such as diagnosing PJI [13].

Using command line tools such as Porechop and Nanofilt enables the removal of adapter sequences and low-quality reads quickly once these programmes have been installed. Adapter sequences are added to tag the genomic DNA for nanopore sequencing but are not part of the query DNA [31]. The adapter sequences protect the query DNA from contamination during sequencing. The pipelines created to perform genomic sequence classification using the command line are easy to use. They result in sequence classification of nanopore data in minutes. If genomic sequence classification is done without removal of these adapter sequences, false classification results are produced [32]. In this study, omitting adapter removal resulted in a high number of false-positive hits for *Escherichia coli*.

BLASTN remains the current gold standard for genomic sequence classification, but Kraken2 is faster. ONT has online classification software for classification, WIMP (What’s in my pot). However, this software requires internet connection, and the query data must be uploaded before analysis [33]. Consequently, WIMP is not suitable for ‘in the field’ projects. MINDS has been developed to overcome this problem, however failed to identify the species of interest in our experience. The BLASTN and Kraken2 databases used for genomic sequence classification can be downloaded onto the computer to improve classification speed and classification can be performed offline.

Currently, a Synovasure test is used intraoperatively to rule out PJI. Whilst useful in identifying the presence of infection, it cannot identify the causative organism and is expensive. Nanopore sequencing using the ‘rapid sequencing kit’ can be performed at a cost of around £200 per patient [3], which is considerably cheaper than a £500 Synovasure test, which can only be used to rule out PJI [34]. Although ONT have a sequencing kit for nanopore sequencing of the 16 s region, it requires a longer sample preparation time so was not chosen for this study [35].

## CONCLUSIONS

This preliminary work suggests that there is clinical utility for using nanopore sequencing technology to identify the bacterial species causative of PJIs at the time of revision surgery and only a short sequencing time (around 15 min) may be necessary. More specimen from patients with PJI will need to be collected and a group of uninfected individuals will be analysed as a control population. We were unable to confirm known antibiotic resistance profiles with such a short sequencing time. However, guidance is given on how this can be achieved in the literature. This will be revisited using a longer sequencing time and/or an alternative library preparation kit from ONT.

**Key Points**

- DNA sequencing using the MinION from Oxford Nanopore Technologies can identify bacterial species of an infected prosthetic implant considerably faster than current methods.
- Antibiotic resistance is challenging to identify and requires additional investigation.

## Supplementary Material

appendix_1_elae008

appendix_2_elae008

appendix_3_elae008

appendix_4_elae008

## CONVENTION

Bacterial names in italics: *Staphylococcus aureus.*

## Author Biographies

**Hollie Wilkinson** is a PhD student with Keele University based at the Robert Jones and Agnes Hunt Orthopaedic Hospital. Her interests include genomics, bioinformatics and diagnostic science.

**Jamie McDonald** is an undergraduate student at Cardiff University, who is undertaking a BSc in biological sciences with an Industrial Placement Year at the Robert Jones and Agnes Hunt Orthopaedic Hospital. Jamie applied for Orthopaedic Institute Funding to study if antimicrobial resistance genes could be identified using a rapid sequencing kit from Nanopore Technologies in infected prosthesis samples. Her interests include genetics, proteomics and multi-omics analysis.

**Helen McCarthy** is a post-doctoral research associate with Keele University based at the Robert Jones and Agnes Hunt Orthopaedic Hospital. Her interests include musculoskeletal biology, cell therapy, histology and genomics.

**Jade Perry** is a post-doctoral research associate with Keele University based at the Robert Jones and Agnes Hunt Orthopaedic Hospital. Her interests include cell therapy, imaging and histology.

**Karina Wright** is a professor in orthopaedics and bioengineering for Keele University based at the Robert Jones and Agnes Hunt Orthopaedic Hospital in Oswestry. Her interests include multi-omics analysis, cell therapy, biomarkers and patient stratification.

**Charlotte Hulme** is a lecturer at Keele University based at the Robert Jones and Agnes Hunt Orthopaedic Hospital in Oswestry. Her interests include multi-omics analysis, cell therapy, biomarkers and patient stratification.

**Paul Cool** is a professor in orthopaedic surgery at Keele University and has a clinical practice at the Robert Jones and Agnes Hunt Orthopaedic Hospital. His interests include diagnostics, genomics, statistics and artificial intelligence.
