# Supplementary figures and images for: Using nanopore sequencing to identify bacterial infection in joint replacements: a preliminary study
Source: Brief Funct Genomics. 2024 Mar 30;23(5):509–16. doi: 10.1093/bfgp/elae008 (PMC11428152; doi:10.1093/bfgp/elae008)

**Appendix 1**

Summary of the quality scores of sequencing data produced from ONT


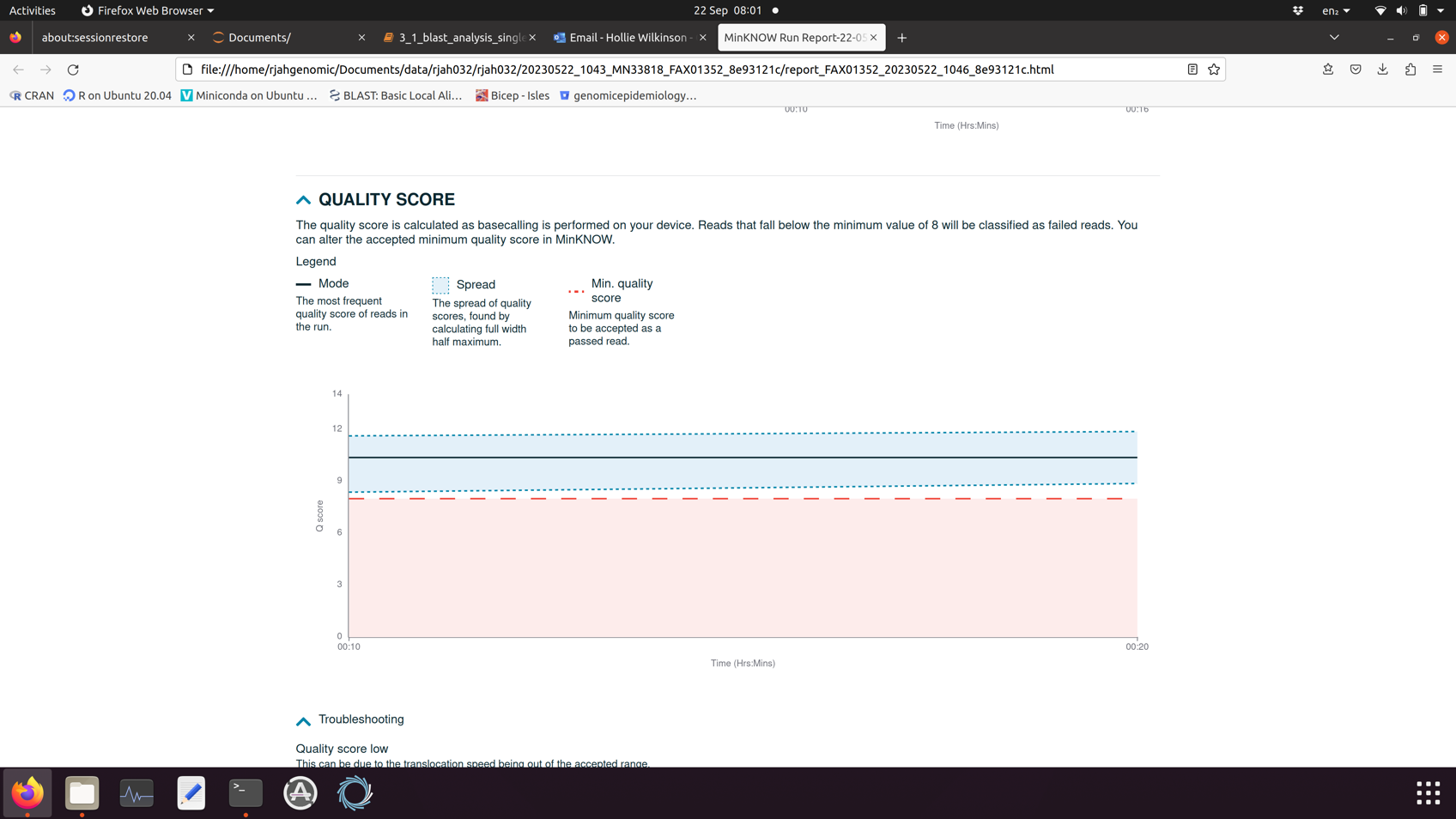

Supplement: appendix_1_elae008 [file appendix_1_elae008.docx]
