# Supplementary material for: Using nanopore sequencing to identify bacterial infection in joint replacements: a preliminary study
Source: Brief Funct Genomics. 2024 Mar 30;23(5):509–16. doi: 10.1093/bfgp/elae008 (PMC11428152; doi:10.1093/bfgp/elae008)
Supplement: appendix_2_elae008 [file appendix_2_elae008.docx]

**Appendix 2**

List of bacterial species used to filter BLAST output:

- *Aeromonas hydrophila*
- *Arcanobacterium haemolyticum*
- *Bacillus*
- *Citrobacter koseri*
- *Citrobacter*
- *Coagulase-negative Staphylococcus*
- *Coagulase-negative Staphylococcus aureus*
- *Corynebacterium amycolatum*
- *Corynebacterium aurimucosum*
- *Corynebacterium propinquum*
- *Corynebacterium striatum*
- *Enterobacter cloacae*
- *Enterobacteriaceae*
- *Enterococcus faecalis*
- *Enterococcus faecium*
- *Escherichia coli*
- *Finegoldia magna*
- *Gemella morbillorum*
- *Granulicatella adiacens*
- *Klebsiella oxytoca*
- *Klebsiella pneumoniae*
- *Micrococcus luteus*
- *Morganella morganii*
- *Mycobacterium fortutium*
- *Propionibacterium acnes*
- *Propionibacterium*
- *Proteus mirabilis*
- *Pseudomonas aeruginosa*
- *Serratia marcescens*
- *Staphylococcus aureus*
- *Staphylococcus condiment*
- *Staphylococcus epidermis*
- *Staphylococcus lugdunensis*
- *Streptococcus agalactiae*
- *Streptococcus dysgalactiae*
- *Streptococcus oralis*
- *Streptococcus pneumoniae*
- *Stapyhlococcus warneri*
- *Staphylococcus capitis*
- *Staphylococcus hominis*
- *Streptococcus viridans*
- *Prevotella*
- *Enterobacter*
- *Streptococcus mitis*
- *Streptococcus milleri*
- *Streptococcus pyogenes*
- *Bacteroides fragilis*
- *Tropheryma whipplei*
- *Ureaplasma urealyticum*
- *Shigella sonnei*
- *Escherichia fergusonii*
- *Neisseria meningitidis*
- *Parvimonas micra*
- *Enterococcus cecorum*
- *Fusobacterium necrophorum*
- *Shigella*
- *Staphylococcus haemolyticus*
- *Staphylococcus caprae*
- *Staphylococcus arlette*
- *Leuconostoc mesenteroides*
- *Acinetobacter baumannii*
- *Pseudomonas aeruginosa*
- *Propionibacterium sp. H456*
- *Klebsiella pneumoniae*
- *Treponema pallidum*
- *Enterococcus faecailis*
