# Supplementary material for: Using nanopore sequencing to identify bacterial infection in joint replacements: a preliminary study
Source: Brief Funct Genomics. 2024 Mar 30;23(5):509–16. doi: 10.1093/bfgp/elae008 (PMC11428152; doi:10.1093/bfgp/elae008)
Supplement: appendix_3_elae008 [file appendix_3_elae008.docx]

**Appendix 3**

Pipeline for genomic sequencing and classification
