# Supplementary material for: Using nanopore sequencing to identify bacterial infection in joint replacements: a preliminary study
Source: Brief Funct Genomics. 2024 Mar 30;23(5):509–16. doi: 10.1093/bfgp/elae008 (PMC11428152; doi:10.1093/bfgp/elae008)
Supplement: appendix_4_elae008 [file appendix_4_elae008.docx]

**Appendix 4**

Software & packages

**R Version**

R - base 4.3.1

askpass   1.1

backports   1.4.1

base           4.2.2

base64enc   0.1-3

bit            4.0.5

bit64     4.0.5

blob      1.2.4

boot      1.3-28

broom   1.0.4

bslib     0.4.2

cachem 1.0.8

callr     3.7.3

cellranger 1.1.0

class     7.3-20

cli       3.6.0

clipr     0.8.0

cluster  2.1.4

codetools 0.2-18

colorspace 2.1-0

compiler  4.2.2

conflicted 1.2.0

cpp11     0.4.3

crayon        1.5.2

curl      5.0.0

data.table 1.14.8

datasets  4.2.2

DBI            1.1.3

dbplyr    2.3.2

digest         0.6.31

dplyr     1.1.2

dtplyr         1.3.1

ellipsis  0.3.2

evaluate  0.21

fansi     1.0.3

farver    2.1.1

fastmap   1.1.1

fontawesome   0.5.1

forcats   1.0.0

foreign        0.8-83

fs       1.6.2

gargle    1.4.0

generics  0.1.3

ggplot2   3.4.2

glue      1.6.2

googledrive   2.1.0

googlesheets  4 1.1.0

graphics 4.2.2

grDevices 4.2.2

grid      4.2.2

gtable    0.3.3

haven     2.5.2

highr     0.10

hms       1.1.2

htmltools 0.5.5

httr      1.4.6

ids       1.0.1

isoband   0.2.7

jquerylib      0.1.4

jsonlite       1.8.4

KernSmooth 2.23-20

knitr     1.42

labeling  0.4.2

**Python Version**

Python 3.11

attrs           19.3.0

backcall           0.1.0

biopython       1.74

bleach       3.1.0

certifi             2019.11.28

cgecore             1.5.6

cycler             0.10.0

decorator         4.4.1

defusedxml       0.6.0

entrypoints       0.3

gitdb               4.0.9

GitPython         3.1.18

importlib-metadata 1.3.0

ipykernel           5.1.3

ipython             7.10.2

ipython-genutils   0.2.0

ipywidgets         7.5.1

jedi               0.15.1

Jinja2             2.10.3

jsonschema         3.2.0

jupyter             1.0.0

jupyter-client     5.3.4

jupyter-console     6.0.0

jupyter-core       4.6.1

kiwisolver         1.1.0

mappy               2.17

MarkupSafe         1.1.1

matplotlib         3.1.1

mistune             0.8.4

mkl-fft             1.0.15

mkl-random         1.1.0

mkl-service         2.3.0

more-itertools     8.0.2

nbconvert           5.6.1

nbformat           4.4.0

notebook           6.0.2

numpy               1.17.4

olefile             0.46

pandas             0.25.3

pandocfilters       1.4.2

parasail           1.1.19

parso               0.5.2

patsy               0.5.1

pexpect             4.7.0

pickleshare         0.7.5

Pillow             6.2.1

pip                 19.3.1

prometheus-client   0.7.1

prompt-toolkit     2.0.9

ptyprocess         0.6.0

Pygments           2.5.2

pyparsing           2.4.5

pyrsistent         0.15.6

pysam               0.14.1

python-dateutil     2.8.1

pytz               2019.3

PyYAML             5.2

pyzmq               18.1.0

qcat               1.1.0

qtconsole           4.6.0

reportlab           3.5.26

scipy               1.3.2

seaborn             0.9.0

Send2Trash         1.5.0

setuptools         42.0.2

six                 1.13.0

smmap               5.0.0

statsmodels         0.10.1

tabulate           0.8.10

terminado           0.8.3

testpath           0.4.4

tornado             6.0.3

traitlets           4.3.3

typing-extensions   4.1.1

wcwidth             0.1.7

webencodings       0.5.1

wheel               0.33.6

widgetsnbextension 3.5.1

zipp               0.6.0

**Unix programs Version**

BLAST 2.13.0

Flye 2.9.2

AMRFinderPlus 3.11.17

ResFinder 4.0

Resistance Gene Identifier 6.0.2
